# Global transcriptional analysis of human FHs 74 Int intestinal epithelial cells after exposure to advanced glycation end products

**DOI:** 10.1371/journal.pone.0331325

**Published:** 2025-09-10

**Authors:** Katharina Schwertner, José Basílio, Karin Hoffmann-Sommergruber, Isabella Ellinger, Sabine Geiselhart

**Affiliations:** 1 Center for Cancer Research, Medical University of Vienna & Comprehensive Cancer Center (CCC), Vienna, Austria; 2 Institute of Pathophysiology and Allergy Research, Center for Pathophysiology, Infectiology & Immunology, Medical University of Vienna, Vienna, Austria; 3 Division of Pharmacology, Department of Pharmacology, Physiology and Microbiology, Karl Landsteiner University of Health Sciences, Krems, Austria; Ewha Womans University, KOREA, REPUBLIC OF

## Abstract

Advanced glycation end products (AGEs) and reactive intermediates, such as methylglyoxal, are formed during thermal processing of foods and have been implicated in the pathogenesis of a series of chronic inflammatory diseases. AGEs are thought to directly interact with the intestinal epithelium upon ingestion of thermally processed foods, but their effects on intestinal epithelial cells are poorly understood. This study investigated transcriptomic changes in human intestinal epithelial FHs 74 Int cells after exposure to AGE-modified human serum proteins (AGE-HS), S100A12, a known RAGE ligand, and unmodified human serum proteins (HS). In contrast to previous studies employing cancer cell lines, RNA sequencing of FHs 74 Int cells treated with AGE-HS did not reveal transcriptional changes associated with increased proliferation, increased expression of tight junction proteins or proinflammatory responses. Surprisingly, neither AGE-HS nor S100A12 treatments resulted in significant differential gene expression at standard analysis thresholds, while unmodified HS induced minor transcriptional changes. Gene set enrichment analysis revealed that AGE-HS treatment induced downregulation of gene sets linked to MYC, interferon responses, and oxidative phosphorylation, as well as pathways related to neurodegenerative diseases such as Alzheimer’s, Parkinson’s, and Huntington’s disease, paralleling some effects observed with S100A12. This is the first global transcriptomic analysis of FHs 74 Int cells and the first unbiased investigation of signaling pathway alterations in intestinal epithelial cells exposed to AGEs. In contrast to previous studies, this analysis did not reveal any significantly differentially expressed genes, thus challenging previous reports of robust AGE-induced inflammatory and proliferative effects and emphasizing the importance of an isolated experimental setting and rigorous endotoxin testing.

## Introduction

The Western diet – typically high in ultra-processed foods, rich in sugars, fats and proteins – has been shown to substantially impact human health. Particularly, its contribution to the increasing prevalence of chronic inflammatory and immune-related diseases has raised considerable concerns in recent years [1,2].

Ultra-processing of foods rich in sugar, fat, and protein results in the formation of a heterogeneous group of compounds known as advanced glycation end products (AGEs). AGEs and reactive precursors such as methylglyoxal (MG) are generated through the Maillard reaction, a non-enzymatic glycation reaction, in which reducing sugars react with free amino groups of proteins, lipids, or nucleic acids [3]. In the last decades, AGEs have gained increasing scientific attention as they have been identified as significant contributors to the pathogenesis of various chronic diseases such as diabetes, atherosclerosis, neurodegenerative diseases, and cancer [3,4].

The intestinal mucosa designates the largest immunological site of the human body and the main interaction site for ingested compounds. Intestinal homeostasis is preserved by a complex interplay of intestinal epithelial cells (IECs), the local immune system, and the microbiome. IECs function as immune sentinels by expressing pattern recognition receptors, such as Toll-like receptors (TLRs) and NOD-like receptors, which detect microbial components and initiate immune signaling cascades [5,6]. IECs regulate immune responses through soluble mediators, which direct dendritic cells and other immune cells to balance tolerance and defense [7] and simultaneously maintain physical barrier integrity via formation of tight junction complexes and secretion of mucus [8]. Chronic stressors can subvert the protective role of IECs and drive pathological immune activation. Dysregulation of mucosal immune responses is a significant factor in the pathogenesis of inflammatory bowel disease (IBD) and the development of food allergies. A compromised barrier function and aberrant immune signaling disrupt oral tolerance, leading to sustained inflammation [9,10].

Consumption of thermally processed foods results in chronic exposure of IECs to dietary AGEs, which can directly interact with cellular receptors in the intestinal epithelium [11,12]. AGEs are thought to primarily signal via RAGE, the receptor for advanced glycation end products [13]. In addition, several other receptors may also play a role in internalization or in the initiation of signaling cascades [14–17]. Notably, CD36, which is expressed in IECs, has been demonstrated to form signaling complexes with other receptors, such as TLRs and integrins [18].

Despite the number of studies that have investigated the effect of AGEs on the intestinal epithelium, conclusive data is still lacking [12]. While AGEs are often considered pro-inflammatory, some studies also suggest they may have anti-inflammatory properties in certain contexts [12,19]. Particularly, there is still a substantial gap in understanding whether AGEs can interact directly with IECs, and if so, which mechanisms and signaling pathways are involved in initiating cellular responses. Moreover, the role of RAGE in IECs interacting with AGEs is not conclusively understood.

Although very sophisticated research models of the human intestine exist, intestinal epithelial cell line models simplify the complex digestive system and thus serve as a valuable tool for basic research. The FHs 74 Int cell line is a robust model of non-transformed IECs [20,21], originally derived from the healthy small intestine of a 3–4 months gestation female patient, exhibiting epithelial-like morphology. FHs 74 Int cells form tight junctions and express proteins such as ZO-1, claudins, and occludin. A recent study showed that FHs 74 Int cells can develop robust barrier properties comparable to those of Caco-2 cells when grown on permeable supports, forming a functional tight monolayer [22]. Furthermore, they have been shown to express RAGE [23], the main receptor for AGEs, capable of inducing signal transduction [24].

The aim of the present study was to examine transcriptomic changes in IECs induced by AGE-modified proteins. Understanding direct molecular effects of AGEs on these cells is crucial for gaining deeper insight into the complex mechanisms underlying their potential role in various intestinal diseases. Therefore, the healthy fetal intestinal epithelial cell line FHs 74 Int was exposed to unmodified human serum proteins (HS), MG-modified human serum proteins (AGE-HS), and S100A12, a known RAGE ligand [25,26], for 1 and 4 hours. Transcriptional changes in FHs 74 Int cells were assessed by RNA-sequencing, and gene expression profiles were used to identify AGE-dependent signaling pathways by gene set enrichment analysis (GSEA).

To our knowledge, this is the first global transcriptomic analysis of FHs 74 Int cells, and this study represents the first unbiased investigation of signaling pathway alterations in IECs derived from healthy tissue exposed to AGEs. This RNA sequencing dataset fills a substantial gap in understanding the interactions between AGE-modified proteins and the intestinal epithelium, thereby providing mechanistic insights into their impact on chronic disease.

## Materials and methods

### Intestinal epithelial cells

Human FHs 74 Int cells were obtained from the American Type Culture Collection (ATCC, CCL-241) and maintained following ATCC instructions. Briefly, cells were grown in T75 flasks (surface activated for adherent cells; TPP, Switzerland) using HybriCare medium (ATCC, 46-X) supplemented with 10% fetal-calf serum (Gibco), 100 U/mL penicillin and 100 µg/mL streptomycin (Gibco), and 30 ng/mL epidermal growth factor (EGF; BioLegend).

### Modification of proteins

Human AB serum (Sigma-Aldrich) was treated with MG (Sigma-Aldrich) at a final concentration (f.c.) of 100 mM for 24 hours at 37°C and dialyzed against PBS (Gibco) using a 3.500 MWCO membrane (Slide-A-Lyzer Cassette, ThermoFisher) [27]. Fluorescence of HS and AGE-HS was measured in triplicate using a Tecan Spark microplate reader (Tecan). Fluorescence intensity was read at 510 nm after excitation at 320 nm [28]. In order to test the modifications that were generated by MG-treatment, we initially tested the procedure by treating bovine serum albumin (BSA) (Sigma-Aldrich). Subsequently, modified BSA (AGE-BSA) was subjected to LC-MS using a high-resolution Q Exactive HF Orbitrap mass spectrometer, and several AGE-specific amino acid modifications were identified (S1A Fig in S1 File). To visualize the grade of aggregation of serum proteins after MG-treatment, SDS-PAGE (10%) and Coomassie Blue staining were performed. Modified proteins were tested for endotoxin levels using a highly sensitive NF-κB driven TLR4 reporter cell line kindly provided by Peter Steinberger (Medical University of Vienna), according to the protocol provided by Radakovics *et al*. [29]. Lipopolysaccharide (LPS; Sigma-Aldrich) was used as a positive control.

### Cell treatment and RNA extraction

FHs 74 Int cells (passage 4) were seeded in 6-well plates at a density of 1.5 x 10^5^ cells/well. Cells were grown for 3 days until they were confluent. Prior to treatment, cells were starved (medium without serum and EGF) for 2 hours and then treated (triplicate) with the respective substances (AGE-HS, f.c. 200 µg/mL; HS, f.c. 200 µg/mL; S100A12, f.c. 200 ng/mL) for 1 and 4 hours, and PBS-treated cells served as a control. Human recombinant S100A12 was purchased from R&D Biosciences. Before and after treatment, the cells were visually monitored under an optical inverted microscope. Cells were washed three times with PBS, harvested in 350 µl of RLT lysis buffer (Qiagen), containing 1% 2-mercaptoethanol, and immediately frozen at −20°C. For the extraction of RNA, samples were thawed, and total RNA was extracted using the RNeasy Mini Kit (Qiagen) including DNase I digestion, according to the manufacturer’s protocol.

### RNA sequencing

Sequencing libraries were prepared from total RNA samples at the Core Facility Genomics, Medical University of Vienna, with the QuantSeq FWD using the UDI V2 protocol (Lexogen). Eleven PCR cycles were performed for library preparation, determined by qPCR according to the manual. Libraries underwent quality control on a Bioanalyzer 2100 (Agilent) with a High Sensitivity DNA Kit to confirm correct insert size and were quantified using Qubit dsDNA HS Assay (Invitrogen). The pooled libraries were sequenced on a NextSeq500 instrument (Illumina) in 1x75bp single-end mode, generating approximately 3.8 million reads per sample. FASTQ format reads were produced using the Illumina bcl2fastq command line tool (v2.19.1.403). Reads were then trimmed and filtered with cutadapt [30] version 2.8 to remove polyA tails, reads with N’s, and bases with a quality of less than 30 from the 3’ ends. After this procedure, 2.2 million reads remained on average. FASTQ reads were aligned to the human reference genome version GRCh38 [31] with Gencode 29 annotations [32] using the STAR aligner [33] version 2.6.1a in 2-pass mode. STAR counted the raw reads per gene.

### Differential expression analysis

The data analysis was performed in RStudio Server version 2024.4.1.748 [34]. We retained only protein-coding genes with a total read count greater than zero and a valid Entrez Gene ID. In cases where multiple gene IDs mapped to the same Entrez ID, we kept the gene ID with the highest overall read count to ensure a unique Entrez ID per gene. Annotation was performed with package AnnotationDbi version 1.66.0 [35] with annotation database org.Hs.e.g.,db version 3.19.1 [36]. Differential expression analysis was performed using DESeq2 version 1.44.0 [37] and Wald test. *P*-values were adjusted using the Benjamini & Hochberg method [38]. Reported log_2_ fold changes (LFC) were obtained by shrinkage with *lfcShrink* function from DESeq2 ashr version 2.2.63 [39]. For the Venn Diagram, only those genes with an adjusted *p*-value < 0.05 and an absolute LFC ± 1 were considered. The R *p*ackages ggplot2 version 3.5.1 [40] and VennDiagram version 1.7.3 [41] were used for data visualization.

### Gene set enrichment analysis

GSEA [42] addresses limitations in traditional differential expression analysis, which often focuses on individual genes with large differences. GSEA detects coordinated but subtle changes across predefined gene sets by aggregating per-gene statistics. Here we used a ranked gene list, ordered by signed −log_10_(*p-*value), where the sign reflects the LFC. This ranking method integrates both the significance and direction of gene expression changes, enabling the identification of meaningful patterns across related genes [43]. The enrichment analysis was performed with clusterProfiler version 4.14.0 [44], which internally utilizes the fgsea method for fast preranked gene set enrichment analysis [45]. Only those gene sets with a minimum size of 10, maximum size of 500, adjusted *p*-value < 0.05 were considered for further analysis. The gensets used were: 1) the Hallmark Geneset collection from Molecular Signatures Database [46], extracted using msigdbr version 7.5.1 [47] and Kyoto Encyclopedia of Genes and Genomes (KEGG) [48–50] which is built in the clusterProfiler package.

## Results

### Preparation of MG-modified HS proteins (AGE-HS)

**Fig 1:**
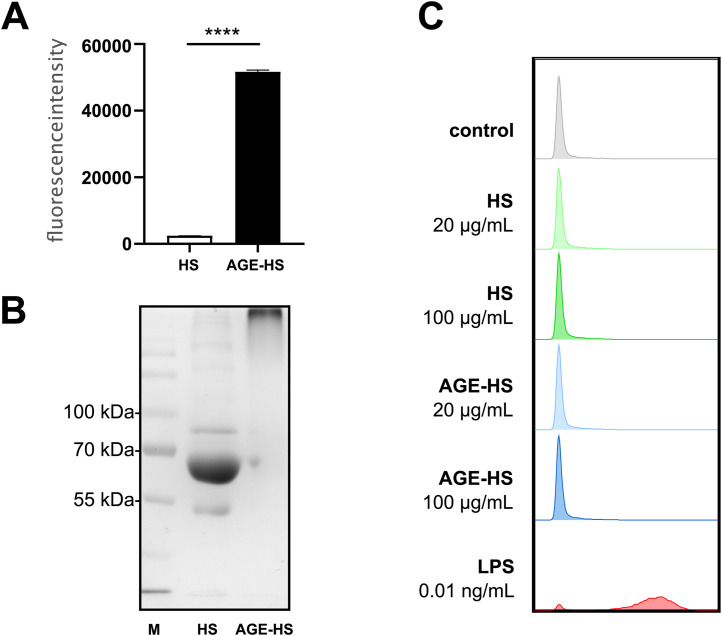
Characterization of MG-modified HS proteins (A) Fluorescence measurement of HS and AGE-HS. **(B)** Coomassie-stained SDS-PAGE of unmodified (HS) and MG-modified (AGE-HS) serum proteins **(C)** Endotoxin testing of samples using a TLR4 reporter cell line.

MG and MG-modified proteins are formed during thermal processing of foods, especially in high-temperature treatment like grilling, frying, or toasting. It has been shown that RAGE specifically recognizes MG-modified proteins [51]. Therefore, human serum proteins were modified by incubation with MG, a highly reactive intermediate that forms MG-hydroimidazolone modifications on arginine residues and carboxyethyl modifications on both lysine Nε-carboxyethyllysine and arginine Nε-carboxyethylarginine residues [52]. Furthermore, fluorescent AGEs such as argpyrimidine are formed [53]. After incubation with MG, HS proteins showed the characteristic browning. AGE-associated fluorescence (excitation 320 nm; emission 510 nm) was measured, and significantly higher fluorescence intensity was detected in MG-modified HS (AGE-HS) compared to unmodified HS (Fig 1A). The MG-modified proteins also showed a high degree of aggregation as visualized by Coomassie-stained SDS-PAGE (Fig 1B).

### The modified protein samples are free of endotoxin

Endotoxins such as LPS bind to TLR4 and activate intracellular signaling cascades such as the NF-κB and MAPK pathways, which lead to the production of pro-inflammatory cytokines. To ensure that the samples are free of endotoxin, we tested them using an NF-κB-based TLR4 reporter cell line that can detect 500 fg/mL LPS [29]. The reporter cells were not activated by any of the tested samples (except LPS, representing the positive control), showing that the samples were free of endotoxin and could be used for cell culture experiments (Fig 1C, S2 Fig in S1 File).

### Differential gene expression

RNA-sequencing was performed to elucidate changes in gene expression in FHs 74 Int cells upon treatment with AGE-HS. Two time points, 1 hour and 4 hours, were chosen to examine immediate and early responses. Additionally, FHs Int 74 were treated with unmodified HS and S100A12, a known ligand of RAGE [26]. PBS-treated cells served as control.

**Fig 2:**
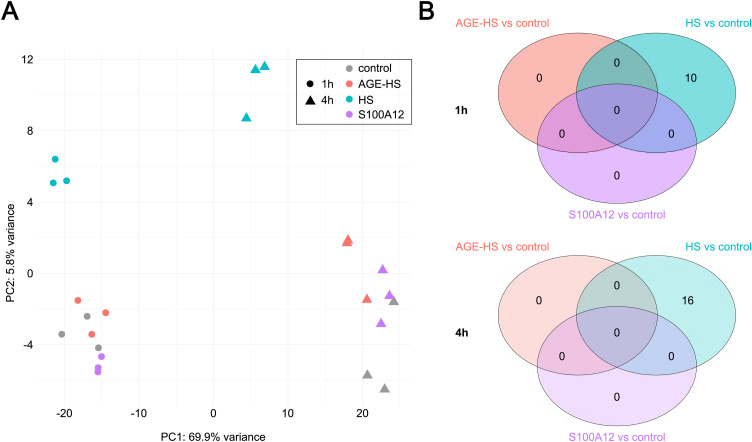
Transcriptional profiling of untreated, AGE-HS-, HS-, and S100A12-treated FHs 74 Int cells. **(A)** Principal component analysis **(B)** Venn diagram of different comparisons.

Principal component analysis (PCA) (Fig 2A) revealed that the primary source of variation was the two time points, as captured by Principal Component 1 (PC1). The secondary source of variation corresponded to the difference between HS-treated cells and those subjected to other treatments. Notably, there was no clear separation observed among AGE-HS, PBS, and S100A2-treated cells. Differentially expressed genes between the treatment groups were identified using DESeq2, employing an LFC cutoff of ±1. Treatment with AGE-HS proteins did not result in any significant changes in gene expression at either time point. Similarly, treatment with S100A12 did not alter gene expression. However, with an LFC of −0.99, right at the cut-off limit, CCN1 was downregulated after 1 hour. As anticipated from the PCA, treatment with HS proteins induced significant changes in gene expression, with 10 and 16 genes differentially expressed after treatment for 1 hour and 4 hours of treatment, respectively (Fig 2B). Differentially expressed genes (adjusted *p*-value < 0.05, no LFC cutoff) after treatment with AGE-HS and S100A12 are shown in S1 Table.

### Gene set enrichment analysis

**Fig 3:**
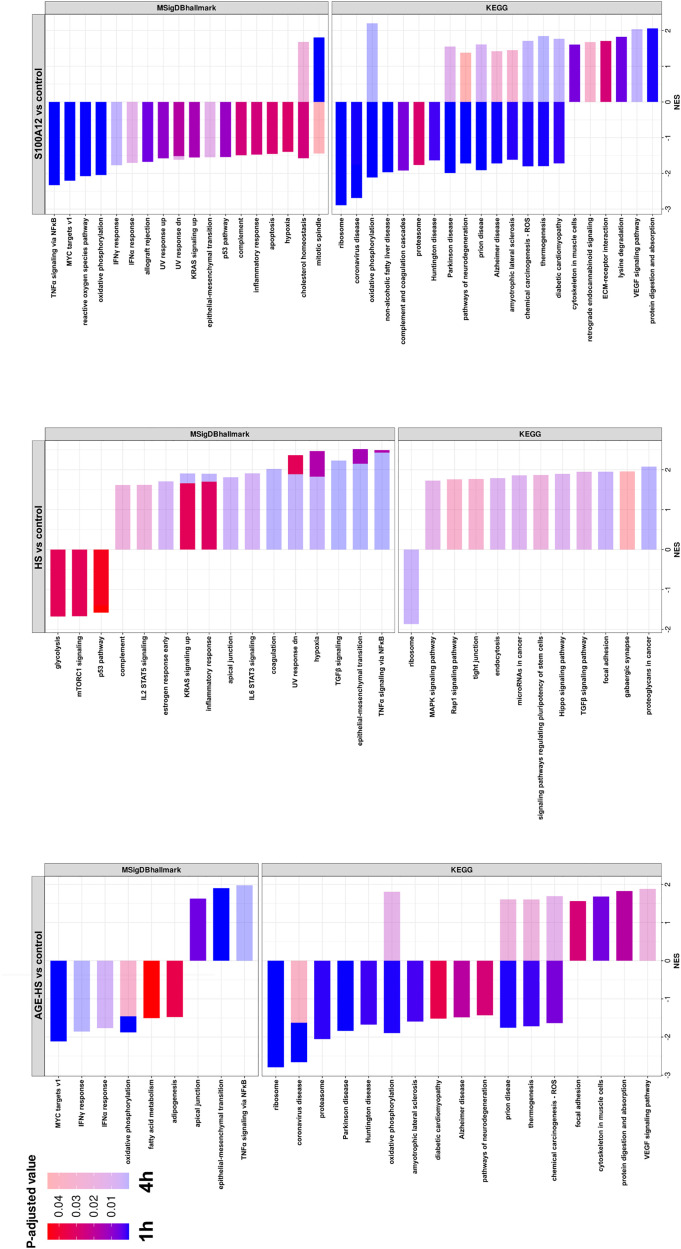
Gene Set Enrichment Analysis. MsigDB and KEGG of FHs 74 Int cells after treatment with AGE-HS, HS, and S100A12. Gene set expression changes after 1h and 4h are shown according to their significance level in strong and light colors, respectively.

To define transcriptional programs driven by treatment of FHs 74 Int cells with HS, AGE-HS, and S100A12, GSEA using the MSigDB Hallmarks and KEGG were performed (Fig 3).

When cells were treated with AGE-HS, GSEA revealed upregulation of the Hallmarks “apical junction”, “epithelial-mesenchymal transition”, and “TNFα signaling via NF-κB”. However, these gene sets were also upregulated in cells treated with HS proteins. Pathways upregulated after treatment with AGE-HS were not found among the upregulated pathways in S100A12-treated cells. In fact, treatment with S100A12 led to the downregulation of “epithelial-mesenchymal transition” and “TNFα signaling via NF-κB” signaling pathways.

Treatment with AGE-HS proteins resulted in significant downregulation of gene sets “MYC targets v1”, “oxidative phosphorylation”, “IFNα response”, and “IFNγ response” as well as “fatty acid metabolism” and “adipogenesis”. Interestingly, some of the gene sets downregulated after treatment with AGE-HS proteins (“MYC targets v1”, “IFNγ response”, “interferon α response”, “oxidative phosphorylation”), were also found among the gene sets downregulated by S100A12 treatment, while these remained unchanged by HS treatment.

GSEA based on the KEGG pathway database revealed several significant findings. Treatment with AGE-HS resulted in upregulation of pathways related to “focal adhesion”, “cytoskeleton in muscle cells”, “protein digestion and absorption”, and “VEGF signaling pathway”. Interestingly, some pathways (“oxidative phosphorylation”, “prion disease”, “thermogenesis”, “chemical carcinogenesis”) were upregulated after treatment with AGE-HS proteins for 1h, while they were subsequently downregulated after 4h of treatment. Remarkably, the same effect was also observed in cells treated with S100A12 (“oxidative phosphorylation”, “prion disease”, “thermogenesis”, “chemical carcinogenesis”). All these pathways (with the exception of “focal adhesion”) were unaffected in HS protein-treated cells. Furthermore, AGE-HS induced downregulation of pathways related to “ribosome”, “coronavirus disease”, “proteasome”, “Parkinson disease”, “Huntington disease”, “amyotrophic lateral sclerosis”, “diabetic cardiomyopathy”, “Alzheimer disease”, and “pathways of neurodegeneration”. All these downregulated pathways were also found among the downregulated pathways of S100A12-treated cells, while only “ribosome” was also affected in HS protein-treated cells.

## Discussion

The trend towards highly processed foods has significantly elevated the consumption of thermally treated products, resulting in chronic exposure of the intestinal mucosa to dietary AGEs. To date, no human intervention studies have been conducted investigating the effects of AGE-modified proteins on human health. Some animal studies, mostly based on healthy rodent models, have addressed this issue, but with contradicting findings. Qu *et al.* showed that dietary AGEs partially increased colon permeability in rats, characterized by reduced gene expression of tight junction components occludin and ZO-1. Furthermore, increased serum LPS levels and altered composition of the colon microbiome were observed [54]. Impaired gut barrier function was also observed in a diabetic mouse model [55]. Contrarily, Chu et al. showed that glycated casein can alleviate intestinal inflammation and improve gut health, suggesting potential protective effects related to glycation [56]. Along with this study, Cao et al. showed that feeding fish protein with low or medium glycation extent resulted in increased ZO-1 and occludin expression, lowered inflammation markers maintaining gut barrier integrity. Interestingly, feeding fish protein with high glycation extent to mice had the opposite effect, leading to increased gut permeability [19]. Nass et al. demonstrated that feeding of AGE-modified proteins to transgenic mice resulted in a transient and systemic activation of the NF-κB pathway in multiple tissues [57]. These *in vivo* studies do not clearly distinguish whether the observed effects, positive or negative, result from direct interaction with IECs or involve other cell types, such as immune or stromal cells.

In contrast, *in vitro* studies using intestinal epithelial cell lines treated with AGEs provide insights into direct cellular responses and underlying mechanisms. Treatment of Caco2BBe cells with glycated casein increased oxidative stress and proliferation and activated NF-κB and Akt signaling [58]. Liang and colleagues stated that treatment with AGEs induced proliferation, invasion, and epithelial-mesenchymal transition via PI3K/Akt signaling pathways in human SW480 colon cancer cells [59]. Chen *et al.* [60] and Deng *et al.* [61] observed increased proliferation of colorectal cancer cells in a RAGE-dependent mechanism. Lin *et al.* demonstrated that treatment of HCT116 colon cancer cells with glycated BSA increased the expression of RAGE and boosted malignancy via MAPK/NF-κB-induced STAT3 and β-catenin expression [62]. Furthermore, Wang *et al.* described upregulation of oncogenic MDM2 as well as degradation of p53 and Rb in a RAGE-dependent manner, after treatment of HCT116 cells with glycated BSA [63].

However, all these *in vitro* studies were performed using cell lines derived from colon cancer. Relying on intestinal cancer cell lines may limit the translational relevance of findings, as these models often exhibit altered genetic and metabolic profiles that do not accurately reflect the behavior of healthy intestinal cells under physiological conditions. Consequently, this discrepancy can result in misleading conclusions about treatment effects and cellular responses.

In this study, a cell line derived from the healthy small intestine was used to overcome these limitations. FHs 74 Int cells develop tight junctions, form an epithelial monolayer, and express RAGE [22,23]. In contrast to previous studies employing cancer cell lines, RNA sequencing of FHs 74 Int cells treated with AGE-modified proteins did not reveal gene expression changes associated with increased proliferation, increased expression of tight junction proteins, or proinflammatory responses. Moreover, differential expression analysis did not reveal any significantly differentially expressed genes at LFC ± 1. This is despite the fact that not only RAGE is expressed in FHs 74 Int cells, but also several other receptors that have been described to bind AGEs and have the potential to trigger signaling cascades through the formation of complexes with other receptors (S3 Fig in S1 File) [17,18].

In contrast, we observed differential gene expression after treatment with unmodified HS proteins. In line with this, exposure of starved IECs to naïve serum proteins induced proliferation and migration, causing upregulation of genes associated with these processes [64,65]. It has been reported that glycation of proteins results in a significant loss of function due to changes in protein conformation, aggregation, and irreversible cross-linking [66,67]. As we have also observed massive protein aggregation after MG-treatment (Fig 1B), we speculate that after glycation, serum proteins no longer fulfill their physiological functions and thus do not induce serum-dependent cellular responses in our model.

S100A12, also known as calgranulin C, was one of the first members of the S100 family confirmed to interact with RAGE and to exert inflammatory effects in a RAGE-dependent manner, as reported by Hofmann *et al.* in 1999 [68]. In airway epithelial cells, S100A12-RAGE binding activated ERK1/2 and NF-κB [69]. In FHs 74 Int cells, however, not only AGE-modified proteins, but also S100A12 did not induce a significant response in gene expression.

Although no significant differences in the expression of individual genes were observed with DESeq2, GSEA revealed some significantly affected pathways in cells treated with AGE-HS. GSEA can capture subtle but coordinated changes in biological functions that may stay undetected when traditional differential expression, based on single-gene analysis is used. GSEA based on MSigDB Hallmarks revealed significant downregulation of gene sets associated with cellular responses to IFNα and IFNγ in AGE-HS-treated cells. This downregulation could reflect a compensatory mechanism to prevent barrier integrity and avoid excessive immune activation. Additionally, expression of a gene set linked to MYC signaling was decreased [70]. Since oxidative stress or inflammatory stimuli can lead to MYC suppression, this could be a protective response to avoid propagating damaged DNA or triggering tumorigenic pathways. As these gene sets were also downregulated in S100A12-treated cells, but not in HS-treated cells, a common signaling mechanism can be assumed.

GSEA based on the KEGG pathway database identified a notable impact on pathways associated with neurodegenerative diseases such as Alzheimer’s, Parkinson’s, or Huntington’s disease after treatment with AGE-HS and S100A12. Interestingly, all these pathways were downregulated after one hour in both treatments. After four hours of treatment, most KEGG pathways related to neurodegenerative diseases were upregulated in S100A12, but not in AGE-HS-treated cells. The association of AGEs as well as different members of the S100 family with neurodegenerative diseases is well documented [71,72]. However, the observed downregulation of neurodegenerative disease pathways in IECs is unlikely to be directly related to these diseases. While neurodegenerative disease-associated pathways are generally linked to neuronal cells, genes of these pathways are also related to general stress responses such as apoptosis, autophagy, and protein degradation. In the context of IECs, their differential expression may not directly indicate a connection to neurodegenerative diseases. We believe that IECs may have initiated a protective mechanism against AGE-induced stress, rather than direct involvement in neurodegenerative pathways.

Our GSEA results show that oxidative phosphorylation, thermogenesis, and chemical carcinogenesis/ reactive oxygen species pathways were transiently downregulated after one hour and upregulated after four hours of AGE-HS and S100A12 treatment. The initial downregulation of pathways related to energy metabolism, thermogenesis, and mitochondrial function might suggest an acute response to AGE-induced damage and oxidative stress. Subsequent upregulation of these pathways after 4 hours could indicate adaptive or compensatory cellular responses. The upregulation of the VEGF signaling pathway further supports this, as VEGF is an inflammatory mediator in IECs, and its signaling mechanisms have previously been linked to RAGE [73,74].

Notably, these effects were absent in HS-treated cells, highlighting the specific impact of glycation. The results of our study clearly differ from those in the existing literature. In this study, we confirmed that AGE modifications were introduced by testing for AGE-associated fluorescence as well as protein aggregation. We also used LC-MS to analyze MG-treated BSA to identify the resulting modifications of the proteins by this treatment (S1 Fig in S1 File).

In contrast to other studies, the samples used in this study were tested for the presence of endotoxins. In line with our observations, Valencia and colleagues reported that endotoxin-free AGE-BSA did not induce inflammatory signals [75]. Reznikov *et al.* even showed that AGEs can only trigger an immune response in the presence of endotoxin. AGE-modified HSA did not induce peripheral blood mononuclear cells to release proinflammatory cytokines [76]. As also noted by Prantner and colleagues [77], samples in other studies were often not tested for the presence of endotoxins. When studying the inflammatory properties of biological macromolecules, it is important to check for possible endotoxin contamination since endotoxins are known to have strong effects on most cell types. Endotoxins are reported to induce oxidative stress and to modulate tight junction permeability via TLR4 signaling [78,79]. In IECs, endotoxins were shown to modulate cell permeability and inflammation [80]. Remarkably, these effects have been attributed to AGEs in previous studies. Even minimal endotoxin contamination could lead to altered and thus misleading results. Alarmingly, endotoxin contaminations can sometimes be detected in commercially available proteins and extracts labelled as endotoxin-free [81,82], or even in plastic blood collection tubes [83,84]. Unfortunately, this confounder is often overlooked.

Finally, FHs 74 Int cells are a very simplified model of the intestinal epithelium. Stem cells, differentiated IECs, stroma, and immune cells are lacking. Especially, the presence of immune cells was shown to be important for AGE-induced signaling. According to Briceno Noriega *et al.,* AGEs can activate the adaptive immune system when they are taken up, processed, and presented by dendritic cells or monocytes [85]. Additionally, Stick *et al.* showed that AGEs trigger inflammatory responses by activation of mast cells [86].

Overall, this study shows that AGE-modified proteins do not induce significant changes in gene expression in FHs 74 Int cells, a model for healthy IECs. GSEA, however, revealed subtle effects that have not yet been described in the literature. We believe that it is important to report these findings to help understand the complexities of AGEs-related signaling. In comparison to existing literature, this study carefully considered endotoxin testing and included a detailed analysis of AGE-modifications. However, there are also some limitations. Although FHs 74 Int cells do form monolayers and have microvilli [22,87], they do not properly represent the differentiated intestinal epithelium. Also, due to the fetal origin of the cells, age-dependent epithelial responses to AGE exposure are missing. Furthermore, cells were grown in 2D monolayers and may not behave as they do in 3D environments. However, this also applies to the *in vitro* studies mentioned above. To gain a more comprehensive understanding of AGE-induced effects in IECs, further investigations are needed. Future studies should focus on more advanced models, such as intestinal organoids or co-culture systems of IECs with immune cells, to better assess the effects of cytokine secretion and the interplay of epithelial cells with stromal and immune cells. While IECs alone may not show significant effects, biologically relevant responses might emerge in more complex systems employing a combination of epithelial cells and immune cells.

## Supporting information

S1 File S1-S3 Figs. S1 Fig Modifications derived by MG treatment. S2 Fig MG-modified samples do not contain endotoxin. S3 Fig Gene expression levels of AGEs-binding receptors.
(PDF)

S1 Table Differentially expressed genes after treatment with AGE-HS and S100A12. (XLSX)
