# Supplementary material for: Global transcriptional analysis of human FHs 74 Int intestinal epithelial cells after exposure to advanced glycation end products
Source: PLoS One. 2025 Sep 10;20(9):e0331325. doi: 10.1371/journal.pone.0331325 (PMC12422490; doi:10.1371/journal.pone.0331325)
Supplement: S1 File — S1 Fig Modifications derived by MG treatment. S2 Fig MG-modified samples do not contain endotoxin. S3 Fig Gene expression levels of AGEs-binding receptors. (PDF) [file pone.0331325.s001.pdf]

Supporting Information

Supporting Figure 1

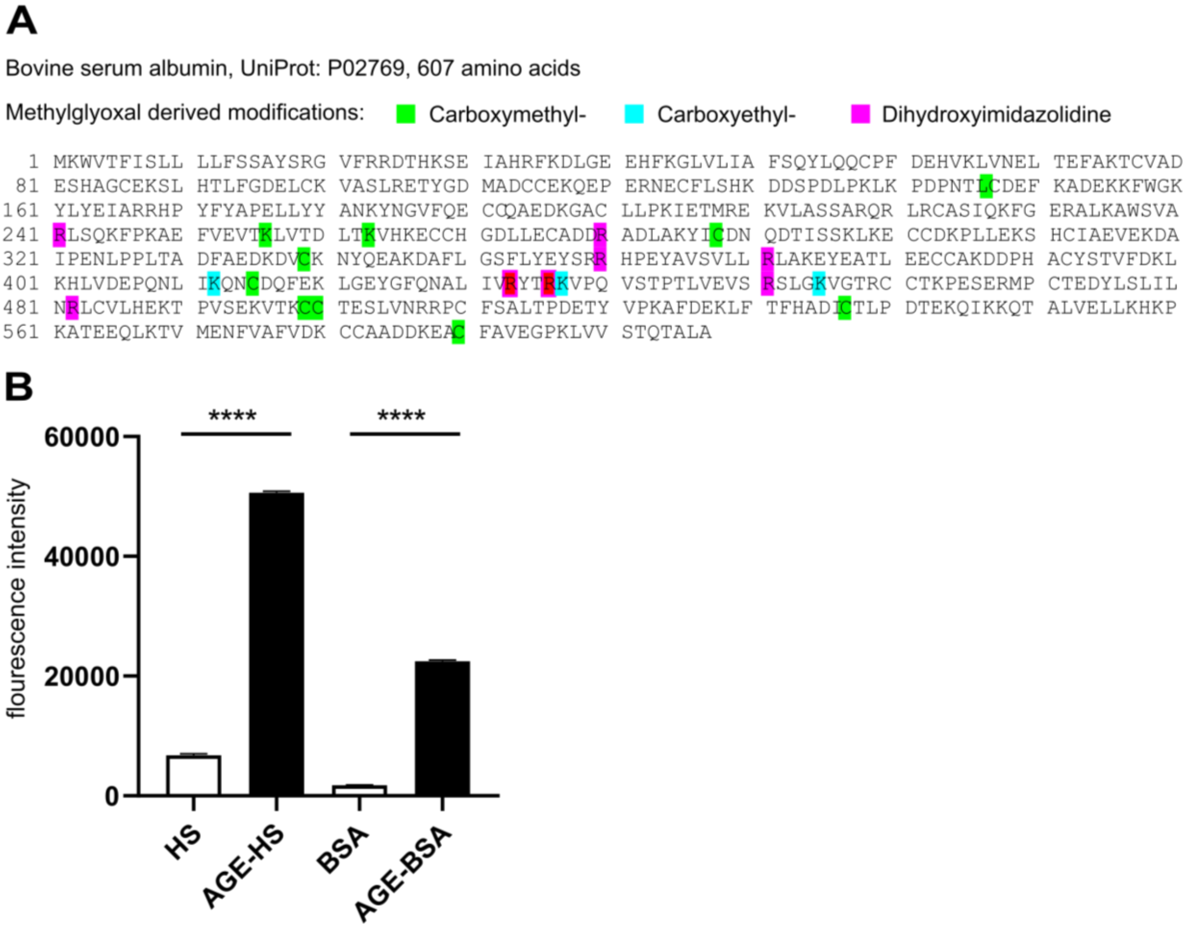

S1 Fig. Modifications derived by methylglyoxal treatment (A) Amino acid modifications on BSA derived by methylglyoxal treatment. **(B)** Fluorescence measurement of HS, AGE-HS, BSA and AGE-BSA

Supporting Figure 2

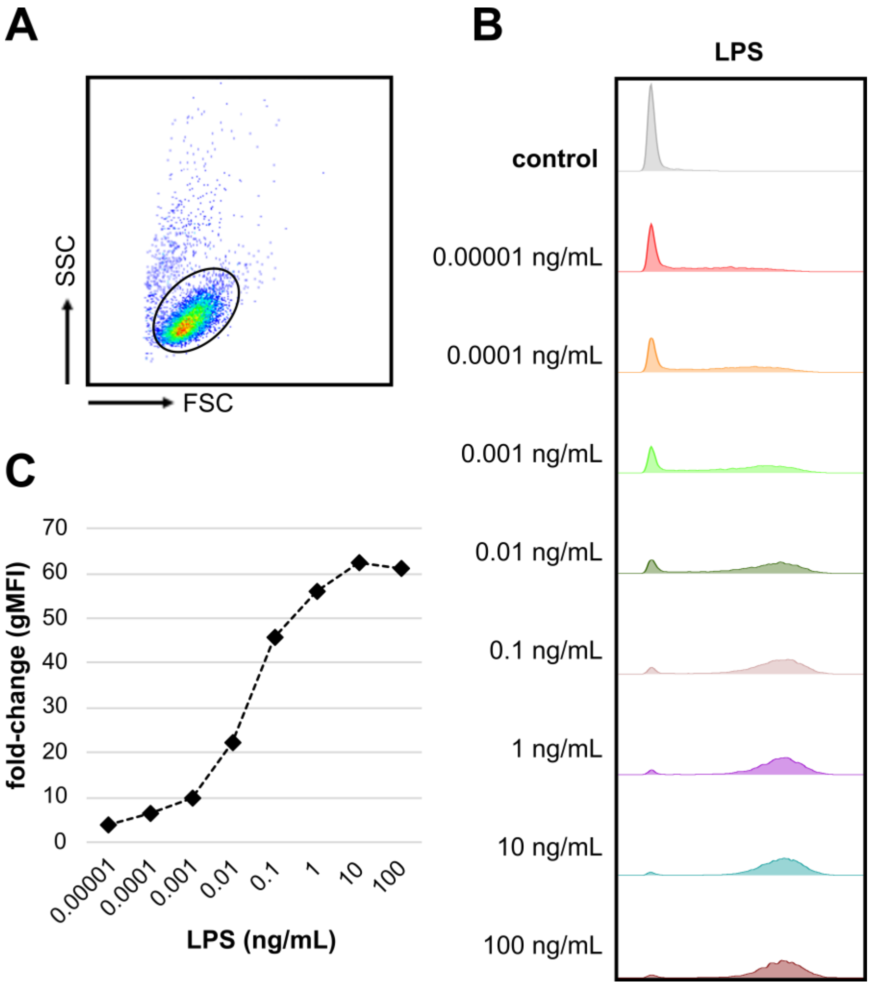

S2 Fig. MG-modified samples do not contain endotoxin. Endotoxin levels of samples were tested with a TLR4 reporter cell line. (**A)** FSC/SSC plot of the reporter cell line, live cells (gate) were used for analysis. (**B, C**) LPS induced a dose-dependent activation of TLR4 reporter cells.

Supporting Figure 3

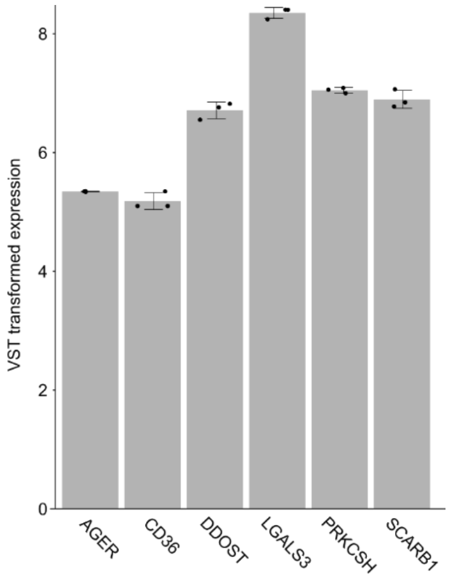

S3 Fig. Gene expression levels of AGEs-binding receptors. RAGE (AGER), CD36, OST-48 (DDOST), Galectin-3 (LGALS3), 80K-H (PRKCSH) and SR-B1 (SCARB1).
